# Interallelic Transcriptional Enhancement as an *in Vivo* Measure of Transvection in *Drosophila melanogaster*

**DOI:** 10.1534/g3.116.032300

**Published:** 2016-08-02

**Authors:** Geoffrey P. Noble, Patrick J. Dolph, Surachai Supattapone

**Affiliations:** * Department of Biochemistry and Cell Biology, Geisel School of Medicine, Dartmouth College, Hanover, New Hampshire 03755; † Department of Medicine, Geisel School of Medicine, Dartmouth College, Hanover, New Hampshire 03755; ‡ Department of Biology, Dartmouth College, Hanover, New Hampshire 03755

**Keywords:** transvection, enhancer, somatic pairing, GAL4-UAS

## Abstract

Transvection—pairing-dependent interallelic regulation resulting from enhancer action *in trans*—occurs throughout the *Drosophila melanogaster* genome, likely as a result of the extensive somatic homolog pairing seen in Dipteran species. Recent studies of transvection in *Drosophila* have demonstrated important qualitative differences between enhancer action *in cis* *vs.* *in trans*, as well as a modest synergistic effect of *cis*- and *trans*-acting enhancers on total tissue transcript levels at a given locus. In the present study, we identify a system in which *cis*- and *trans*-acting GAL4-UAS enhancer synergism has an unexpectedly large quantitative influence on gene expression, boosting total tissue transcript levels at least fourfold relative to those seen in the absence of transvection. We exploit this strong quantitative effect by using publicly available UAS-shRNA constructs from the TRiP library to assay candidate genes for transvection activity *in vivo*. The results of the present study, which demonstrate that *in trans* activation by simple UAS enhancers can have large quantitative effects on gene expression in *Drosophila*, have important new implications for experimental design utilizing the GAL4-UAS system.

The nuclear genome is often pictured to function as a linear arrangement of nucleotides grouped into genes and regulatory elements operating more or less locally *in cis* . However, physical interactions between distant genomic sites, including interactions between distinct chromosomes, are increasingly understood to play important roles in eukaryotic genome function (Cavalli and Misteli 2013). For example, *in trans* interactions between distinct chromosomes have been shown to play a critical role in mammalian genomes during the process of X-inactivation (Masui *et al.* 2011) and the monogenic expression of olfactory receptors (Lomvardas *et al.* 2006).

The paired somatic genomes of *Drosophila melanogaster* and other Dipteran species are a striking example of nuclear organization in which there is widespread physical interaction between maternal and paternal homologous chromosomes (McKee 2004; Metz 1916; Stevens 1908). That the physical pairing of homologous somatic chromosomes in *Drosophila* could have functional consequences was hypothesized over 100 years ago (Stevens 1908), but genetic interaction between physically paired alleles at the same genomic locus was first demonstrated in 1954 by Lewis (1954), who established the term transvection to describe such interactions.

Classically, transvection has been characterized in relatively circumscribed settings in which genetic complementation between two mutant alleles at the same locus can be eliminated by the introduction of chromosomal rearrangements (Lewis 1954). However, the relatively recent development of site-directed transgenesis technologies (Fish *et al.* 2007; Markstein *et al.* 2008) has opened up vast new possibilities for the study of transvection phenomena. Using such technologies, it was recently shown that transvection appears to be a pervasive feature of the *Drosophila* genome, with transcriptional activation *in trans* occurring at essentially all loci tested, independent of enhancer identity, hypothetical pairing sequences, and even strict homology between paired genetic elements (Bateman *et al.* 2012a; Blick *et al.* 2016; Mellert and Truman 2012). A recent study has also demonstrated transvection-like phenomena in budding yeast (Zhang and Bai 2016).

Despite these significant recent advances, transvection remains poorly understood at the mechanistic as well as functional level. One interesting observation made in prior studies of transvection in *Drosophila* has been the detection of quantitatively significant increases in transcription at loci for which transcriptional activation can be driven by regulatory elements that are both *in cis* and/or *in trans* (Bateman *et al.* 2012a; Gibson *et al.* 1999; Goldsborough and Kornberg 1996; Lum and Merritt 2011). Though the effect sizes observed have been modest (Bateman *et al.* 2012a; Gibson *et al.* 1999; Lum and Merritt 2011) or difficult to quantify (Goldsborough and Kornberg 1996), previous authors have speculated that quantitative changes in gene expression as a result of transvection could play important roles in normal and/or pathological genome function. Indeed, somatic chromosomal pairing appears to drive dysregulated gene expression in at least one type of human neoplasm (Koeman *et al.* 2008).

In addition to affecting gene expression quantitatively, transcriptional activation *in trans* also appears to be qualitatively different from transcriptional activation *in cis* (Bateman *et al.* 2012a; Blick *et al.* 2016; Mellert and Truman 2012). Recent studies have shown that *in trans* transcriptional activation occurs stochastically and with variable intensity among neighboring cells within a tissue, while activation *in cis* occurs predictably and at consistent levels within those same tissues (Bateman *et al.* 2012a; Blick *et al.* 2016; Mellert and Truman 2012). These qualitative differences suggest that *in cis* and *in trans* transcriptional activation may be mechanistically different, and raise the interesting question of whether they might utilize different sets of regulatory or effector molecules.

To date, very few molecular factors required for transvection have been identified. Transvection is thought to require the physical proximity of homologous chromosomes, which is maintained by specific cellular factors (Hartl *et al.* 2008), and high throughput screens recently undertaken in *Drosophila* cell culture have identified numerous genes that affect somatic chromosomal pairing (Bateman *et al.* 2012b; Joyce *et al.* 2012). However, such screens do not assess the functional role that identified pairing factors might play in transvection, and an efficient method for assaying chromosomal pairing factors for transvection activity *in vivo* has not yet been developed.

In the present study, using a *Drosophila* model of neurodegeneration, we have identified conditions under which transvection between allelic GAL4-UAS-driven transgenes produces a dramatic (approximately fourfold) increase in transcriptional efficacy that is readily detected by semiquantitative RT-PCR and Western blot. We find that quantitatively similar interallelic increases in transcription can be caused by publicly available UAS-tagged transgenes, including those from the TRiP shRNA library (Ni *et al.* 2011). Finally, by using interallelic enhancement of transcription as a sensitive functional measure of transvection, we utilize TRiP shRNA constructs to test a panel of candidate genes for transvection activity *in vivo*.

## Materials and Methods

### Fly stocks and husbandry

Flies carrying the UAS-mouse (Mo)PrP insert at *attP2* have been previously described (Murali *et al.* 2014). The GAL4 driver lines w^1118^;P{ChAT-GAL4.7.4}/CyO,P{sevRas1.V12}FK1, w*;P{GAL4-elav.L}3, and y^1^ w*;P{tubP-GAL4}LL7/TM3, Sb^1^ Ser^1^, referred to as Cha-GAL4, Elav-GAL4, and α-tubulin-GAL4, respectively, were obtained from the Bloomington *Drosophila* Stock Center (BDSC) (Bloomington, IN). Cha-GAL4 drives UAS-tagged transgene expression in cholinergic neurons at all developmental stages (Salvaterra and Kitamoto 2001), while Elav-GAL4 drives transgene expression in all postmitotic neurons as well as in certain neuroblasts and embryonic glial cells (Berger *et al.* 2007; Robinow and White 1988). Flies were maintained at 25°, 70% relative humidity on standard *Drosophila* cornmeal, yeast, molasses, and agar medium with methylparaben as a mold inhibitor.

### Construction of plasmids and generation of transgenic fly lines

The vector pUASTattB MoPrP was generated previously (Murali *et al.* 2014). To avoid the introduction of mutations into the pUASTattB (Bischof *et al.* 2007) targeting vector, the MoPrP insert was transferred as a *Bgl*II/*Xho*I restriction fragment into the cloning vector pCombo3 (Mike Scott, UCSF) and site-directed mutagenesis performed using the GeneTailor system (Invitrogen, Grand Island, NY) to create pCombo3 P101L, Q171R, and D177N MoPrP constructs. The P101L, Q171R, and D177N MoPrP inserts were then cloned into pUASTattB as *Bgl*II/*Xho*I fragments and the plasmid sequence confirmed by sequencing prior to phiC31-integrase-mediated insertion at *attP2* and *attP40* performed by Genetic Services, Inc. (Cambridge, MA). Isogenic lines carrying UAS-P101L, Q171R, and D177N MoPrP were isolated, balanced, and maintained using standard *Drosophila* genetic techniques.

### Climbing assays

Locomotor function of flies was assessed using an assay similar to that described previously (Ganetzky and Flanagan 1978; Gavin *et al.* 2006; Le Bourg and Lints 1992) and performed at room temperature (22°). Briefly, groups of 15 male flies were collected and allowed to recover from CO_2_ anesthesia for at least 12 hr (at 25°) before being transferred, without anesthesia, into fresh assay vials containing fly media. Flies were allowed to acclimatize for at least 10 min in the assay vial before being tested. To measure locomotor function, flies were knocked to the bottom of the vial and the number climbing 4 cm above the surface of the media within 30 sec was counted. Each group of flies was tested in triplicate and the mean number of flies passing the assay was recorded for that group. At least six independent groups of 15 flies were tested in this way for each genotype, and the mean and standard deviation of the number of flies passing the assay was taken. These values were then normalized to give a percent passing rate for each genotype, which is reported below. Finally, it was noticed that flies with poor locomotor function often displayed improved climbing activity with successive trials, so for all groups of flies tested, six pretrials were performed without a break, followed by the three experimental trials, also without a break. All assays were performed on male flies aged 5 d at 25° after eclosion.

### Preparation of fly head homogenates for protein quantification by Western blotting

Under light CO_2_ anesthesia and using a fresh razor blade, 20 male fly heads were collected and transferred into 50 μl ice-cold lysis buffer (0.5% v/v Triton X-100, 0.5% w/v deoxycholate, 10 mM NaCl, and 50 mM Tris, pH 7.5) in a 0.2 ml glass Kontes micro tissue grinder. A smooth suspension was generated by several strokes of the glass pestle and debris pelleted by a 1 min centrifugation at ∼9500 rcf (relative centrifugal force). 40 μl of the resulting supernatant was mixed with 40 μl of ice-cold 2× SDS-PAGE loading buffer and the sample boiled at 95° for 5 min, then frozen at −80° before electrophoresis and Western blotting. All samples were taken from male flies aged 5 d at 25° after eclosion.

SDS-PAGE was performed as described previously (Deleault *et al.* 2007). After electrophoresis, proteins were transferred to a methanol-charged PVDF membrane using a Hoefer wet transfer apparatus set at a constant current of 1 A for 2 hr. After electroblotting, membranes were blocked for 1 hr at 4° in 15% w/v powdered skim milk dissolved in TBST. The following primary antibodies were used at the indicated dilutions: anti-PrP, mAb 27/33 (Deleault *et al.* 2012) at 1:15,000 in TBST; anti-GFP, JL-8 (Clontech, Mountainview, CA) at 1:10,000 in TBST + milk; anti-kinesin heavy chain, and AKIN01 (Cytoskeleton, Denver, CO) at 1:10,000 in TBST + milk. Membranes were incubated in primary antibody overnight at 4° then washed four times with TBST and incubated for 1 hr at 4° in HRP-conjugated sheep-anti-mouse (GE Biosciences, Piscataway, NJ) or goat-anti-rabbit (Bio-Rad, Hercules, CA) secondary antibody prior to four TBST washes and detection by enhanced chemiluminescence. MoPrP expressed in *Drosophila* appears as a quadruplet of ∼20–25 kDa M*_r_* (Gavin *et al.* 2006). For quantification of MoPrP expression, all four bands were measured in total.

### RNA extraction and semiquantitative RT-PCR

Under light CO_2_ anesthesia and using a fresh razor blade, 30 male fly heads were collected and transferred into 250 μl ice-cold Trizol reagent (Life Technologies, Carlsbad, CA), then frozen at −80° for no more than 1 wk prior to RNA isolation. Total RNA was isolated using the Direct-zol RNA MiniPrep Kit (Zymo Research, Irvine, CA) modified with an in-solution DNase digestion. Briefly, after washing the MiniPrep column with the provided RNA Wash Buffer, 100 μl of DNase digestion solution [∼1 U/ μl RQ1 RNase-free DNase (Promega, Madison, WI) in provided buffer] was added to the column and centrifuged for 30 sec at 500 rcf into an RNase-free 1.5 ml tube. Both column and flow-through were incubated at 37° for 30 min, followed by collection of residual digestion solution in the column matrix by a brief maximal speed centrifugation. The collected ∼100 μl of digestion solution was mixed with 300 μl RNA Binding Buffer (Zymo Research) and 400 μl 100% ethanol, and the column was reloaded and carried through the remaining wash and elution steps. Purity and concentration of the isolated RNA was measured by spectrophotometry.

cDNA synthesis was performed using an oligo dT primer (Roche, Indianapolis, IN) and M-MLV reverse transcriptase (Life Technologies) in reactions containing Protector RNase Inhibitor (Roche). Amplification of cDNA for transcripts encoding PrP or RpL32 was performed simultaneously using 20 cycles of PCR and the following primer pairs: 5′-GGTGGTGGTGACCGTGTGCTGCTT-3′, 5′-CGAACCTTGGCTACTGGCTGCTG-3′ and 5′-AGGCCCAAGATCGTGAAGAA-3′, 5′-TTGTGCACCAGGAACTTCTTGAA-3′, respectively. Samples were stained directly with SybrGold (Life Technologies) at a 1:1000 final dilution and electrophoresed on 1% w/v TBE agarose gels prior to detection by UV transillumination. Under the conditions used, the intensity of the respective amplified bands correlated approximately linearly with the amount of cDNA template added over a 2^6^-fold range spanning the highest and lowest band intensities observed.

### Statistical analysis

Unless otherwise stated, comparisons were performed using one-way ANOVA with Tukey’s HSD post-test. All statistical analyses were performed with GraphPad Prism 6 (GraphPad Software, Inc., La Jolla, CA). Standard deviations are shown as indicated by error bars. *P* < 0.05 was considered statistically significant; * indicates *P* < 0.05, ** *P* < 0.01, *** *P* < 0.001, **** *P* < 0.0001, and ns denotes not significant.

### Data and reagent availability

UAS-PrP fly strains are available upon request. The authors state that all data necessary for confirming the conclusions presented in the article are represented fully within the article.

## Results

While utilizing the GAL4-UAS system in *Drosophila* to model prion protein (PrP)-induced neurodegeneration, we have observed that certain transgenic lines express a neurodegenerative phenotype when the UAS-PrP transgene is homozygous, but not when it is hemizygous, at a given locus (Gavin *et al.* 2006). In these transgenic lines, the neurodegenerative phenotype seen in homozygous UAS-PrP flies is accompanied, and presumably caused, by a dramatic increase in PrP protein levels disproportionate to a twofold increase in UAS-PrP gene dosage (Gavin *et al.* 2006).

**Figure 1:**
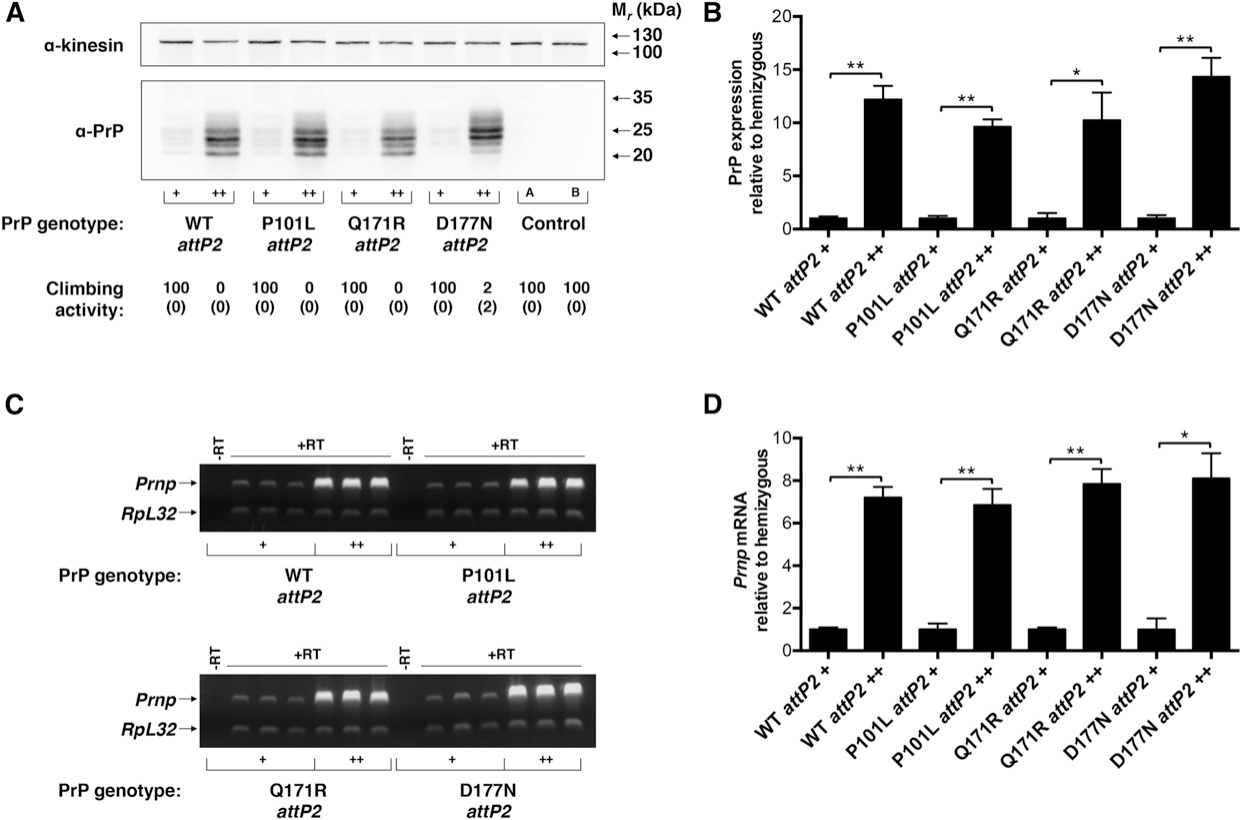
Homozygosity of UAS-PrP at *attP2* increases *Prnp* transcriptional efficacy. Four different UAS-PrP constructs, representing wild-type, pathogenic (P101L and D177N), and protective (Q171R) PrP sequences were inserted at *attP2* and driven by Cha-GAL4. (A) Western blot of fly head homogenates from flies hemi- and homozygous for UAS-PrP constructs at *attP2* (+, hemizygous; ++, homozygous). Experiment was performed in triplicate with a representative blot shown. Climbing activity of the various lines is shown below the corresponding lane of the Western blot and expressed as percent passing the climbing assay (see *Materials and Methods*) with standard deviation shown below in brackets. Control A is driver alone; control B is UAS-PrP alone. (B) Densitometry of Western blot signals in (A) and associated replicates. PrP signals were normalized to the kinesin loading control and then to the respective hemizygous UAS-PrP *attP2* lines. (C) Semiquantitative RT-PCR performed on fly head mRNA of hemi- and homozygous *attP2* UAS-PrP constructs driven by Cha-GAL4 (+, hemizygous; ++, homozygous). *Prnp* and *RpL32* transcripts were coamplified from total cDNA, and each lane represents a biological replicate. Control samples, labeled –RT, represent PCR reactions of input RNA without addition of RT or oligo dT primer during the cDNA synthesis step. (D) Densitometry of RT-PCR signals in (C). For each sample, the ratio of *Prnp* to *RpL32* mRNA levels was taken and then normalized to the average value for the respective hemizygous UAS-PrP *attP2* line. Error bars in (B) and (D) represent standard deviations, with *n* = 3 for each group; ns denotes not significant, * *P* < 0.05, and ** *P* < 0.01, two-tailed Student’s *t*-test. cDNA, complementary DNA; mRNA, messenger RNA; PrP, prion protein; RT, reverse transcriptase; RT-PCR, reverse transcription polymerase chain reaction; WT, wild-type.

To better characterize this phenomenon, we used the site-directed phiC31 integrase system (Fish *et al.* 2007; Markstein *et al.* 2008) to generate transgenic *Drosophila* lines expressing wild-type, pathogenic (P101L and D177N) (Hsiao *et al.* 1990; Jackson *et al.* 2009), and protective (Q171R) (Geoghegan *et al.* 2009; Kaneko *et al.* 1997) PrP variants at the widely used *attP2* locus (Markstein *et al.* 2008) (Figure 1). Unexpectedly, we found that, independent of the misfolding propensity of the UAS-PrP variant expressed, each transgenic line was exquisitely sensitive to UAS-PrP gene dosage at *attP2*; hemizygous UAS-PrP flies passed a simple climbing assay at a rate of 100% and were phenotypically normal, while homozygous UAS-PrP flies of the same line displayed dramatic locomotor defects, with essentially no flies passing the climbing assay (Figure 1A). Moreover, these locomotor defects were accompanied by a 10- to 14-fold increase in PrP protein expression in homozygous UAS-PrP flies relative to hemizygous flies of the same line (Figure 1, A and B). To understand the mechanism of PrP accumulation in these homozygous transgenic lines, we performed semiquantitative RT-PCR on fly head RNA extracts (Figure 1C). Interestingly, we found that PrP mRNA levels were increased approximately seven- to eightfold in homozygous flies relative to hemizygous flies of the same line (Figure 1, C and D), which accounts for the majority of PrP accumulation in these animals and suggests that homozygosity at *attP2* is associated with increased transcriptional efficacy of the UAS-PrP transgene. For all subsequent experiments we focused on the Q171R PrP variant, which we refer to simply as PrP.

**Figure 2:**
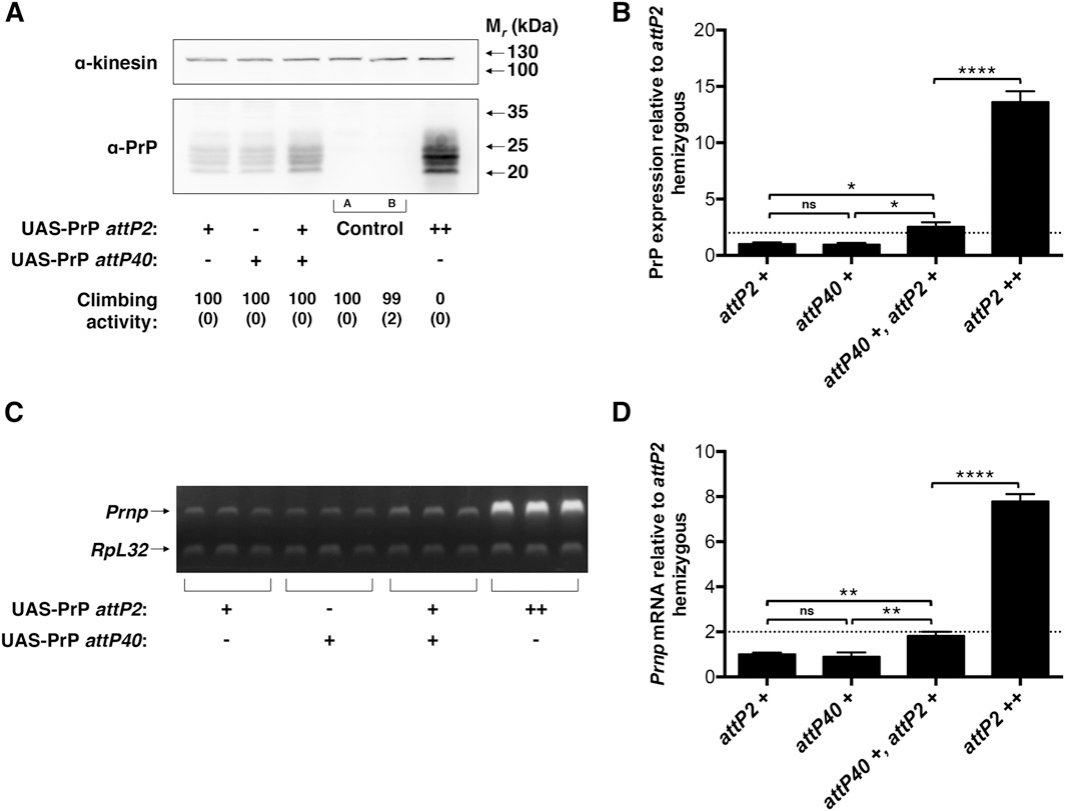
Allelic interaction between UAS-PrP transgenes is required for enhanced *Prnp* transcriptional efficacy. UAS-PrP inserted at *attP2* and/or *attP40* was expressed using the Cha-GAL4 driver. (A) Western blot of fly head homogenates (+, hemizygous; ++, homozygous at the indicated locus). Note that PrP is independently expressed at approximately equal levels from *attP2* and *attP40*. Experiment was performed in triplicate with a representative blot shown. Climbing activity of each line is indicated below the corresponding lane of the Western blot and expressed as percent passing the climbing assay (see *Materials and Methods*) with standard deviation shown below in brackets. Control A is driver alone; control B is UAS-PrP alone. (B) Densitometry of Western blot signals in (A) and associated replicates. PrP signals were normalized to the kinesin loading control and then to the hemizygous *attP2* UAS-PrP line. (C) Semiquantitative RT-PCR performed on fly head mRNA of *attP2* and *attP40* UAS-PrP constructs driven by Cha-GAL4 (+, hemizygous; ++, homozygous at the indicated locus). *Prnp* and *RpL32* transcripts were coamplified in the same PCR reactions, and each lane represents a biological replicate. (D) Densitometry of RT-PCR signals in (C). For each sample, the ratio of *Prnp* to *RpL32* mRNA levels was taken and then normalized to the average value for the hemizygous *attP2* UAS-PrP line. Error bars in (B) and (D) represent standard deviations, with *n* = 3 for each group; ns denotes note significant, ** *P* < 0.01, and **** *P* < 0.0001. Dotted line emphasizes a twofold increase in expression relative to hemizygous. mRNA, messenger RNA; PrP, prion protein; RT-PCR, reverse transcription polymerase chain reaction.

Previous investigations in *Drosophila* have demonstrated that the pairing of *cis*- and *trans*-acting enhancers can synergistically increase transcription at a given genomic locus (Bateman *et al.* 2012a; Gibson *et al.* 1999; Goldsborough and Kornberg 1996; Lum and Merritt 2011), but the effect size reported has been modest (Bateman *et al.* 2012a; Gibson *et al.* 1999; Lum and Merritt 2011) or difficult to quantify (Goldsborough and Kornberg 1996). In these prior studies, mutual transcriptional enhancement between paired alleles has been thought to represent one manifestation of transvection. To test whether the dramatic sensitivity to PrP gene dosage observed in our system (Figure 1) might be a pairing-dependent (*i.e.*, transvection) effect, we created a transgenic *Drosophila* line expressing PrP on a different chromosome, at the *attP40* genomic locus. We found that UAS-PrP transgene insertion at the original *attP2* locus (third chromosome) and at the new *attP40* locus (second chromosome) each yielded almost identical PrP expression levels (Figure 2A, left two lanes and Figure 2B, left two groups). The functional equivalence of these two loci for UAS-PrP expression allowed us to then examine the effect of doubling UAS-PrP gene dosage using copies of UAS-PrP at allelic *vs.* nonallelic loci (Figure 2). We found that, when UAS-PrP transgenes were expressed at nonallelic loci, PrP protein expression was additive and locomotor behavior was normal (Figure 2A, three left lanes). In contrast, when UAS-PrP transgenes were expressed at the same *attP2* genomic site, protein expression was synergistic and flies demonstrated profound locomotor defects (Figure 2A, third lane *vs.* sixth lane). We found analogous results at the mRNA level (Figure 2B), suggesting that homozygosity of UAS-PrP alleles increases the transcriptional efficacy of each UAS-PrP transgene by approximately fourfold (Figure 2D). Synergistic expression of allelic UAS-PrP transgenes was also observed at the *attP40* locus (Supplemental Material, Figure S1). Expression at *attP40* was driven by Elav-GAL4 rather than Cha-GAL4 due to the technical requirement of a third chromosome driver.

**Figure 3:**
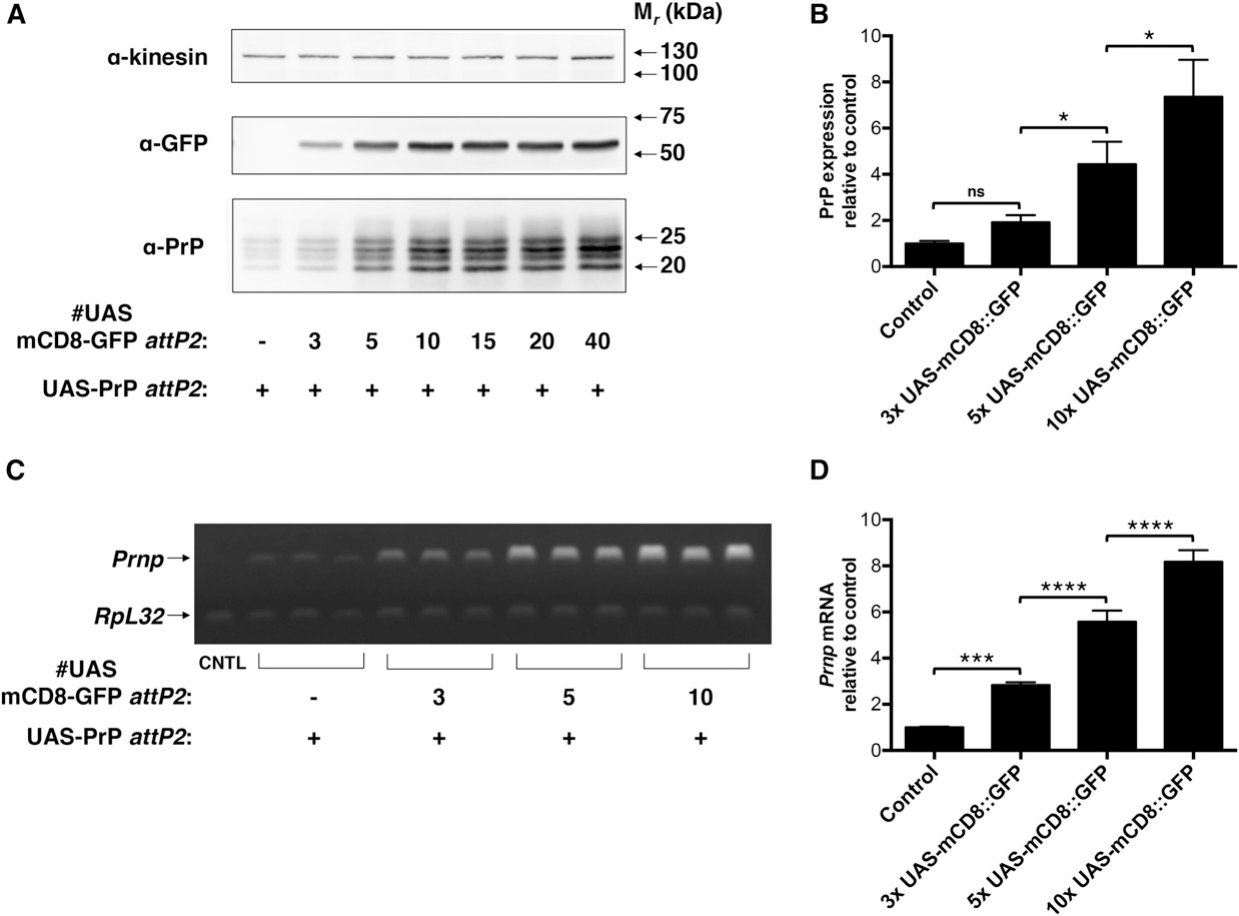
Increasing UAS enhancer strength at the *attP2* locus *in trans* increases *attP2* UAS-PrP expression. UAS-mCD8::GFP constructs with variable UAS number were expressed at *attP2 in trans* to UAS-PrP, with expression driven by Cha-GAL4. (A) Western blot of fly head homogenates. Experiment was performed in triplicate with a representative blot shown. (B) Densitometry of Western blot signals in (A) and associated replicates. PrP signals were normalized to the kinesin loading control and then to the hemizygous *attP2* UAS-PrP line lacking UAS-mCD8::GFP *in trans*. (C) Semiquantitative RT-PCR performed on fly head mRNA. *Prnp* and *RpL32* transcripts were coamplified in the same PCR reactions, and each lane represents a biological replicate. CNTL is of mRNA prepared from UAS-PrP flies that do not express GAL4. (D) Densitometry of RT-PCR signals in (C). For each sample, the ratio of *Prnp* to *RpL32* mRNA levels was taken and then normalized to the average value for the *attP2* UAS-PrP line lacking UAS-mCD8::GFP *in trans*. Error bars in (B) and (D) represent standard deviations, with *n* = 3 for each group; ns denotes not significant, * *P* < 0.05, *** *P* < 0.001, and **** *P* < 0.0001. Control sample, CNTL; GFP, green fluorescent protein; mRNA, messenger RNA; PrP, prion protein; RT-PCR, reverse transcription polymerase chain reaction.

To further characterize the phenomenon of interallelic transcriptional enhancement, we next used the series of *attP2* UAS-mCD8::GFP lines created by Pfeiffer *et al.* (2010), which carry a varying number of UAS elements upstream of the mCD8::GFP reporter construct, to test: a) Whether enhancement of PrP expression could result from the interaction of UAS-PrP and a non-PrP UAS-driven transgene at the same genomic locus, and b) whether the degree of *in trans* enhancement of PrP transcription depends upon the strength of the UAS enhancer on the paired transgene (Figure 3). We found that, indeed, UAS-mCD8::GFP substantially boosted UAS-PrP expression at the protein and mRNA levels when both transgenes were paired at the *attP2* locus (Figure 3, A and C). In addition, this *trans*-acting transcriptional enhancement was approximately proportional to the number of UAS elements on the paired UAS-mCD8::GFP transgene (Figure 3, B and D), though expression of both mCD8::GFP and PrP appeared to saturate for those lines with mCD8::GFP enhancers containing more than 10 UAS elements (Figure 3A). We believe that the above experiments provide compelling evidence that observed interallelic transcriptional enhancement of UAS-PrP expression is a manifestation of transvection.

**Figure 4:**
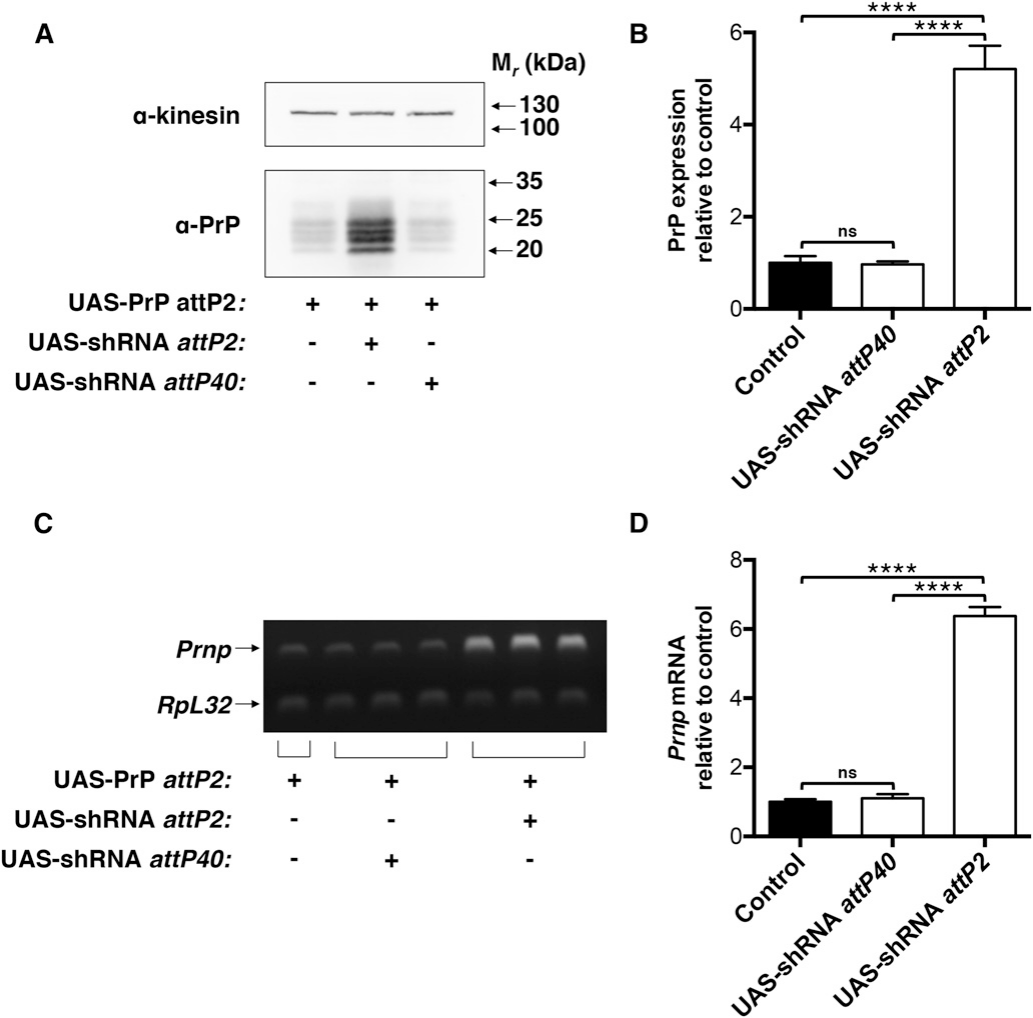
Control UAS-shRNA constructs from the *Drosophila* TRiP library enhance UAS-PrP transcriptional efficacy *in trans*. In flies expressing UAS-PrP from *attP2* driven by Cha-GAL4, an shRNA hairpin against GFP was expressed at the paired *attP2* or at the unpaired *attP40* locus to assess the suitability of the TRiP library as an *in vivo* screening tool for transvection activity. (A) Western blot of fly head homogenates. Experiment was performed in triplicate with a representative blot shown. (B) Densitometry of Western blot signals in (A) and associated replicates. PrP signals were normalized to the kinesin loading control and then to the hemizygous *attP2* UAS-PrP line lacking UAS-shRNA. (C) Semiquantitative RT-PCR performed on fly head mRNA. *Prnp* and *RpL32* transcripts were coamplified in the same PCR reactions, and each lane represents a biological replicate. (D) Densitometry of RT-PCR signals in (C). For each sample, the ratio of *Prnp* to *RpL32* mRNA levels was taken and then normalized to the average value for the *attP2* UAS-PrP line lacking UAS-shRNA. Error bars in (B) and (D) represent standard deviations (*n* = 2 for UAS-PrP alone mRNA quantification, *n* = 3 for all other groups); ns denotes not significant and **** *P* < 0.0001. GFP, green fluorescent protein; mRNA, messenger RNA; PrP, prion protein; RT-PCR, reverse transcription polymerase chain reaction; shRNA, short hairpin RNA; TRiP, Transgenic RNA Interference Project.

Having identified PrP expression as a sensitive measure of cross talk between allelic enhancers in our system, we next sought to determine whether such a measure could be used to identify or study factors required for chromosomal pairing and/or transvection *in vivo*. A powerful tool available for the *in vivo* dissection of biological processes in *Drosophila* is the TRiP library, a genome-scale collection of UAS-shRNA constructs inserted at either the *attP2* or *attP40* sites (Ni *et al.* 2011). To determine whether TRiP constructs could be used in combination with our UAS-PrP lines to study chromosomal pairing and transvection, we obtained TRiP lines expressing identical UAS-shRNA hairpins against an irrelevant gene (GFP) inserted at either *attP2* or *attP40*, and crossed these lines to our *attP2* UAS-PrP line (Figure 4). As predicted, we observed a robust increase in PrP protein and mRNA levels, approximately five- to sixfold, when UAS-PrP and UAS-shRNA were paired at *attP2*, but not when UAS-shRNA was expressed from the distant *attP40* site (Figure 4, A–D).

**Figure 5:**
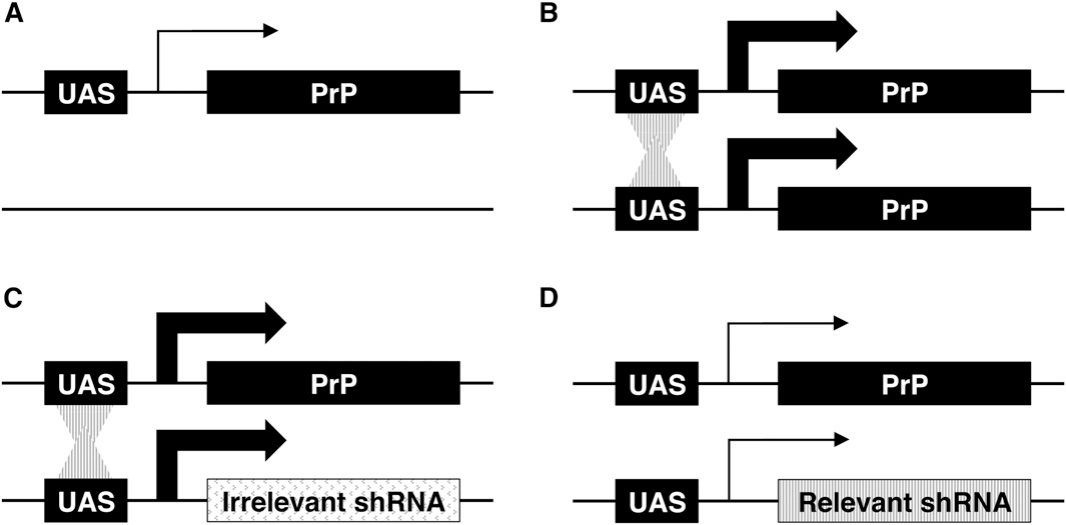
Schematic of an *in vivo* assay for transvection activity based upon interallelic transcriptional enhancement. All transgenes are inserted at the *attP2* locus and the size of the arrow represents the rate of transcription at the associated locus. (A) Hemizygous UAS-PrP is expressed at a low level. (B) Homozygous UAS-PrP is expressed at a high level due to the *in trans* interaction between UAS enhancers at the same locus. (C) UAS-PrP in combination with UAS-shRNAs targeting genes lacking transvection activity (irrelevant shRNA) also leads to high level PrP expression due to the preserved interaction between UAS enhancers *in trans*. (D) UAS-PrP in combination with UAS-shRNAs targeting genes with transvection activity (relevant shRNA) interfere with the *trans* interaction between UAS enhancers, reducing PrP expression. The degree to which target gene knockdown impacts transvection can be simply quantified in this system by a Western blot of PrP levels. PrP, prion protein; shRNA, short hairpin RNA.

Taking our results together, we next designed an experiment to test candidate genes for transvection activity *in vivo*, which is depicted schematically in Figure 5. Briefly, TRiP UAS-shRNA constructs integrated at the *attP2* site were expressed *in trans* to UAS-PrP. In this experiment, hairpins that do not target genes required for transvection (Figure 5C) would be expected, through the presence of their UAS enhancers, to boost UAS-PrP transcription at the allelic *attP2* locus five- to sixfold, as seen with control anti-GFP hairpins (Figure 4). On the other hand, hairpins targeting essential transvection factors would be expected to reduce or eliminate interallelic transcriptional enhancement (Figure 5D). In this system, the degree to which target gene knockdown impacts transvection could be simply quantified by a Western blot for PrP protein levels.

**Figure 6:**
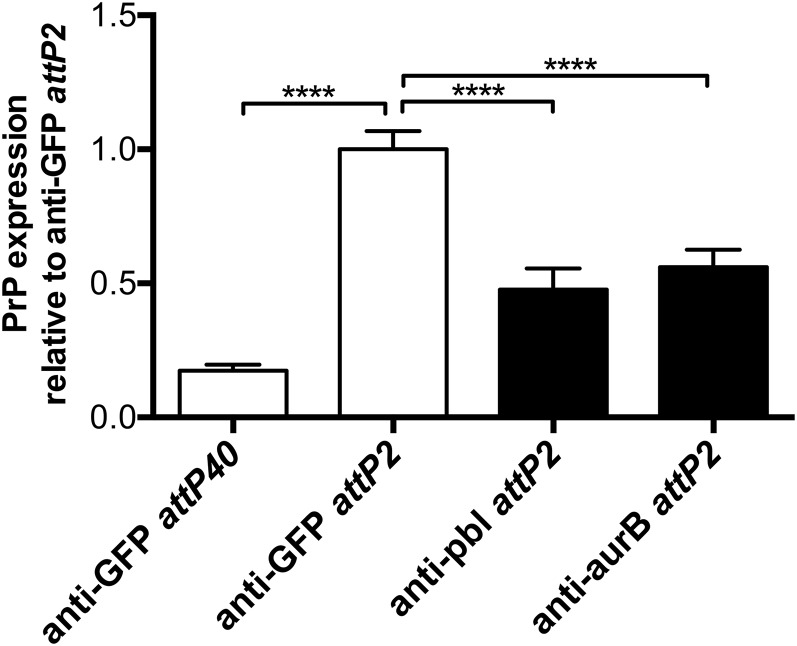
Expression of shRNAs against two putative chromosomal pairing factors, *pbl* and *aurB*, reduces interallelic transcriptional enhancement *in vivo*. Flies expressing UAS-PrP *in trans* to UAS-shRNAs directed against 16 different genes thought to be involved in chromosomal pairing were generated. Transgene expression was driven in cholinergic neurons by Cha-GAL4 and interallelic enhancement of PrP expression was measured by Western blot of fly head homogenates. PrP expression levels were normalized to the level seen in the presence of an anti-GFP control hairpin *in trans*. Error bars represent standard deviations, with *n* = 3 for experimental samples and *n* = 4 for control samples; **** *P* < 0.0001. We found that shRNAs against *pbl* and *aurB* significantly decreased interallelic enhancement of PrP expression. GFP, green fluorescent protein; PrP, prion protein; shRNA, short hairpin RNA.

Therefore, we obtained TRiP UAS-shRNA constructs targeting 25 different genes identified previously in *in vitro* screens (Bateman *et al.* 2012b; Joyce *et al.* 2012) or limited *in vivo* studies (Fritsch *et al.* 2006; Nguyen *et al.* 2015) as playing a role in somatic homolog pairing (Table 1), which is believed to be a prerequisite for transvection. shRNAs targeting 16 of these pairing factors produced viable adult flies when expression was driven by Cha-GAL4 (Table 1), allowing us to test whether the targets of those shRNAs played a role in transvection-mediated interallelic transcriptional enhancement. Interestingly, we found that hairpins targeting just two of these 16 candidate genes, *pbl* and *aurB*, significantly reduced interallelic transcriptional enhancement of UAS-PrP expression *in vivo* (Figure 6). Given that *in vivo* knockdown validation is challenging when shRNA expression is limited to select cells within a tissue of interest, we attempted to get around this problem by expressing each of the shRNAs assayed for transvection-modifying activity ubiquitously with an α-tubulin-GAL4 driver. With the exception of the hairpins against *Hsc70Cb* and *su(Hw)*, all of the shRNAs that produced viable adults when expressed with Cha-GAL4 were lethal when expressed with α-tubulin-GAL4 (Table 1), implying successful knockdown of an essential endogenous gene product. As a control to demonstrate that shRNA sequence specificity was required for this lethality, α-tubulin-GAL4-driven expression of an shRNA against the nonessential *w* gene produced viable adult flies with white eyes (Table 1 and G. P. Noble, unpublished data).

**Table 1:** Testing viability of chromosomal pairing factor knockdown *in vivo*

		Adult Viability with shRNA Expression	
Gene	BDSC shRNA Stock Number	Cha-GAL4 > shRNA	α-tubulin-GAL4 > shRNA
*CG2469*	33,736	**−**	N/a
*chb*	34,669	**−**	N/a
*Dhc64C*	36,698	**−**	N/a
*Klp61f*	33,685	**−**	N/a
*Nlp*	33,688	**−**	N/a
*shtd*	38,531	**−**	N/a
*SkpA*	32,991	**−**	N/a
*slmb*	33,986	**−**	N/a
*Tlk*	33,983	**−**	N/a
*Arp1*	32,032	**+**	**−**
*aurB*	28,691	**+**	**−**
*cal1*	41,716	**+**	**−**
*CG7236*	27,505	**+**	**−**
*CKIα*	25,786	**+**	**−**
*Cul1*	29,520	**+**	**−**
*Det*	51,837	**+**	**−**
*E(bx)*	33,658	**+**	**−**
*fzy*	40,933	**+**	**−**
*Hsc70Cb*	33,742	**+**	**+**
*ida*	34,552	**+**	**−**
*Lis-1*	35,043	**+**	**−**
*Nc73EF*	33,686	**+**	**−**
*pbl*	28,343	**+**	**−**
*polo*	33,042	**+**	**−**
*su(Hw)*	34,006	**+**	**+**
*w*	33,623	**+**	**+**

shRNAs from the TRiP library against the listed genes, which have been identified in previous studies as playing a role in somatic homolog pairing, were obtained from the BDSC. shRNA expression was driven in cholinergic neurons with the Cha-GAL4 driver, or ubiquitously with the α-tubulin-GAL4 driver. shRNA against the *w* gene was included as a positive control. shRNA, short hairpin RNA; BDSC, Bloomington *Drosophila* Stock Center; −, no viable adult flies recovered; N/a, cross not performed; +, viable adult flies recovered; TRiP, Transgenic RNA Interference Project.

## Discussion

Transvection—the *in trans* transcriptional activation or repression of an allele by regulatory elements present on a physically paired homologous allele—has recently been shown to be a pervasive feature of the *Drosophila* genome (Bateman *et al.* 2012a; Chen *et al.* 2002; Mellert and Truman 2012), likely due to the existence of somatic homolog pairing in *Drosophila* and other Dipteran species (Hartl *et al.* 2008; McKee 2004; Metz 1916; Stevens 1908). Although the existence of transvection throughout the *Drosophila* genome is now firmly established, relatively little is known about this genetic regulatory mechanism and its implications for genome function. With the development of site-directed transgenesis technologies (Fish *et al.* 2007; Markstein *et al.* 2008), it is now possible to readily and precisely study transvection phenomena *in vivo*.

In the present study, we have identified a strong interallelic interaction in which *in trans* transcriptional activation boosts overall transcription at the *attP2* locus by at least fourfold relative to levels achieved in its absence (Figure 2). Although we have not performed chromosomal rearrangements in order to formally define this interallelic interaction as transvection, the fact that the effect is observed only when the transgenes tested are inserted at homologous loci (Figure 2 and Figure 4) suggests strongly that transvection is involved. This, to our knowledge, is the largest relative increase in transcription at a tissue level that has been attributed to transvection to date, and a striking example in which apparent synergism, as opposed to additivity, between *cis*- and *trans*-acting enhancers has been observed (Figure 2).

Quantitative increases in transcription at a given locus that can be attributed to the interallelic interaction of enhancers have been reported in prior studies (Bateman *et al.* 2012a; Gibson *et al.* 1999; Goldsborough and Kornberg 1996; Lum and Merritt 2011). To the best of our knowledge, the largest such increases have been observed at the *Gpdh* (Gibson *et al.* 1999) and *Men* (Lum and Merritt 2011) loci, at which up to approximately 100% of an enhancer’s activity *in cis* could be directed toward transcriptional activation *in trans*. However, in these two prior studies, *trans*-acting enhancer activity was measured using alleles with disrupted promoters *in cis*, which may have exaggerated the *trans*-acting effect (Geyer *et al.* 1990; Martinez-Laborda *et al.* 1992). In another study at the *Ubx* locus, quantitatively significant pairing-dependent increases in transcription were also observed, but the relative magnitude of the observed effect is challenging to assess due to widely different activities of the paired *cis*- and *trans*-acting enhancers that were studied (Goldsborough and Kornberg 1996).

To the best of our knowledge, the clearest prior quantitative comparisons to the present study, which examines the effect of pairing on transcription at the tissue level of intact, fully functional alleles, are provided by Bateman *et al.* (2012a) and Schoborg *et al.* (2013). In Bateman *et al.* (2012a), tissue transcript levels of a single allele at the 53F locus were increased modestly (∼15%) when combined with a second functional allele *in trans*. In Schoborg *et al.* (2013) homozygous alleles exhibited at most a ∼50% increase in transcriptional efficacy compared to hemizygous alleles. In contrast, in the present study, we observe that interaction between fully functional alleles *in trans* produces at least a ∼300% increase in the transcriptional output of each allele when measured at the tissue level (Figure 2).

The magnitude of the effect observed in the present study, many-fold higher than in previous reports, raises the question: why is the action of *in trans* enhancers (*i.e.*, transvection) so strong in the present system? We have shown that the effect does not appear to be a unique feature of the Cha-GAL4 driver or of the *attP2* locus, given that interallelic transcriptional enhancement was also observed using the *attP40* locus and the Elav-GAL4 driver (Figure S1). We have further shown that the magnitude of the effect is highly dependent on the strength of the enhancer located *in trans* (Figure 3) and is independent of strict homology between the paired alleles (Figure 3 and Figure 4). These results provide confirmation of the findings of Mellert and Truman, who demonstrated that the presence of GAL4 bound to UAS *in trans* to a functional promoter is sufficient to drive transvection (Mellert and Truman 2012). These results also support the recent findings of Blick *et al.* (2016), who showed that the propensity of many endogenous *Drosophila* enhancers to act *in trans* is related to the strength of the enhancer’s activity *in cis*, and is markedly increased by enhancer multimerization (Blick *et al.* 2016). Of note, the exogenous UAS enhancer used to drive PrP expression in the present study contains five tandem repeats of the GAL4 transcription factor binding site (Bischof *et al.* 2007), which may be one reason why it is an unusually efficient *in trans* activator.

Whether transcriptional activation *in cis* and *in trans* are mechanistically similar or different is a question that remains unanswered. That transcription can be so dramatically increased at a particular locus as a result of transvection, as is seen in the present study, seems to suggest that the mechanisms of transcriptional activation *in cis* and *in trans* are fundamentally different. Indeed, recent studies have already shown that transcriptional activation *in trans* occurs stochastically (Bateman *et al.* 2012a; Blick *et al.* 2016; Mellert and Truman 2012) and at there can be widely varying levels among the cells of a particular tissue, with occasional “jackpot” cells exhibiting intense *in trans* activation (Bateman *et al.* 2012a; Blick *et al.* 2016). Thus, the strong apparent synergism seen between allelic enhancers in the present study (*e.g.*, Figure 2) may represent the summation of infrequent jackpot expression events within the studied tissues, as opposed to a fourfold enhancement of gene expression across all PrP-expressing cells. Alternatively, *in trans* transcriptional activation could be mechanistically similar to *in cis* transcriptional activation, with the relative efficiency of each being determined largely by physical proximity of enhancer-promoter elements at a given locus in a given cell. This latter possibility is supported by the observation that homolog anti-pairing factors inhibit transvection (Hartl *et al.* 2008). However, it is not readily apparent how such a model would account for the significant interallelic enhancer synergism seen in the present study.

Taking the view that *in cis* and *in trans* transcriptional activation could be mechanistically different, it is not yet clear what might be occurring at a molecular level to make them so. One intriguing possibility is that these two transcriptional programs utilize different sets of effector molecules. To the best of our knowledge, no simple methods for identifying or screening for such transvection factors have yet been proposed. The system used in the present study, which a) provides a sensitive and readily quantified readout of transvection (Figure 2), and b) can be manipulated using a variety of publicly available *Drosophila* transgenes (Figure 3 and Figure 4), provides a unique opportunity to study the molecular basis of *in trans* transcriptional activation.

To that end, we utilized our UAS-PrP expression system in combination with shRNAs from the TRiP library (Ni *et al.* 2011) to assay candidate genes for transvection activity *in vivo* (Figure 5). We focused our investigation on genes previously implicated in homologous chromosome pairing, which is thought to be a prerequisite for transvection. Of 25 candidate genes known to be involved in chromosomal pairing (Bateman *et al.* 2012b; Fritsch *et al.* 2006; Joyce *et al.* 2012; Nguyen *et al.* 2015) for which shRNA lines were available, 16 produced viable adult flies when shRNAs against the gene in question were expressed within cholinergic neurons using the Cha-GAL4 driver (Table 1). Interestingly, of these 16 candidate genes that were subsequently assayed for transvection activity, shRNA-targeting of only two of the genes, *pbl* and *aurB*, led to a marked reduction in interallelic transcriptional enhancement (Figure 6).

It is important to acknowledge that both *pbl* and *aurB* play a role in the cell cycle (Bateman *et al.* 2012b; Joyce *et al.* 2012), and that knockdown of cell cycle genes may affect cell viability and/or cell fate. In the present assay, it is not possible to distinguish between decreased PrP expression due to inhibition of interallelic transcriptional enhancement *vs.* the loss of Cha-GAL4-expressing neurons secondary to *pbl* or *aurB* depletion.

Similarly, it is important to acknowledge that pairing of heterochromatin and euchromatin is known to occur with different frequencies in cell culture (Williams *et al.* 2007), and some authors have suggested that this observation may be a reflection of distinct hetero- and euchromatin pairing mechanisms. The majority of the genes tested in our screen (Table 1) were initially identified as playing a role specifically in heterochromatin pairing (Bateman *et al.* 2012b; Joyce *et al.* 2012). However, Joyce *et al.* (2012) have shown extensive overlap between pairing activities for heterochromatin and euchromatin. Indeed, several of the pairing factors targeted in the present screen (Table 1) have previously been shown to affect both hetero- and euchromatin pairing in cell culture (Joyce *et al.* 2012; Nguyen *et al.* 2015), suggesting that a lack of euchromatin pairing activity is unlikely to be responsible for the fact that only two of the 16 putative pairing factors targeted in the present study appear to affect transvection-mediated interallelic transcriptional enhancement (Figure 6).

Furthermore, to the best of our knowledge, it has not previously been determined whether Cha-GAL4 drives UAS-tagged transgene expression in mitotic cells of the neuronal lineage, though Cha-GAL4 is known to be expressed at all developmental stages (Salvaterra and Kitamoto 2001). Expression of shRNAs in predominately postmitotic cholinergic neurons may be one reason for the discrepancy between the *in vivo* screen results reported here and prior *in vitro* studies using cycling cultured cells.

Unfortunately, validation of shRNA knockdown by RT-PCR or Western blot in the present study is technically very challenging, due to the fact that shRNAs are only expressed in a subset of cells within the studied tissue. As one way around this problem, we attempted to express each of the shRNAs assayed for transvection-modifying activity ubiquitously with an α-tubulin-GAL4 driver (Table 1). Of note, with the exception of the hairpins against *Hsc70Cb* and *su(Hw)*, all of the shRNAs that produced viable adults when expressed with Cha-GAL4 were lethal when expressed by the ubiquitous α-tubulin-GAL4 driver (Table 1), suggesting successful knockdown of an essential endogenous gene product. Given the limited number of appropriate, nonoverlapping TRiP constructs available targeting each candidate gene, we unfortunately cannot exclude the potential for off-target effects causing the observed lethality. However, as a control to demonstrate that some shRNA sequence specificity was required for the lethal effect, α-tubulin-GAL4-driven expression of an shRNA against the nonessential *w* gene produced viable adult flies with white eyes (Table 1 and data not shown).

Understanding the limitations of interpretation highlighted above, and under the assumption that pairing factor knockdown was likely successful in our proof of principle screen, the fact that transvection-mediated interallelic transcriptional enhancement was relatively robust to perturbation in the present study suggests the interesting possibility that transvection may be less dependent on the process of homolog pairing than previously thought. Alternatively, homolog pairing may itself be more robust *in vivo* than in *Drosophila* cell culture. Regardless, this preliminary study suggests that an analogous system—one that similarly takes advantage of robust interallelic transcriptional enhancement—might be useful in a reverse genetics approach to potentially identify as yet unknown molecular factors involved in transvection.

Due to the widespread existence of transvection within the *Drosophila* genome, prior investigators have cautioned against the use of multiple transgenes inserted at the same genomic locus when designing *in vivo* experiments (Mellert and Truman 2012). Those prior warnings were based largely on qualitative changes in gene expression that may result from transvection. The present study, which demonstrates that *in trans* activation by simple UAS enhancers can have large quantitative effects on gene expression in *Drosophila*, should be taken as a further cautionary tale for those designing experiments utilizing the GAL4-UAS system; not only should it be assumed that paired UAS enhancers boost overall transcription levels, one should also consider that unpaired UAS enhancers may confound experimental results by driving increased expression of endogenous genes, either *in trans* at the paired wild-type locus, as initially suggested by Mellert and Truman (2012) or locally *in cis*, as has been seen with the GMR enhancer (Hay *et al.* 1997).

In the present study, we have identified an example of transvection in *Drosophila* highlighting the significant quantitative effect that interallelic enhancer interactions can have on gene expression at the tissue level. We have utilized this system to test previously identified chromosomal pairing factors for transvection activity *in vivo*. Our findings add new data to the growing body of work suggesting that transvection may profoundly affect genome function in *Drosophila* and other organisms.

## 

## Supplementary Material

Supplemental Material
