# Supplementary material for: Interallelic Transcriptional Enhancement as an in Vivo Measure of Transvection in Drosophila melanogaster
Source: G3 (Bethesda). 2016 Aug 2;6(10):3139–48. doi: 10.1534/g3.116.032300 (PMC5068936; doi:10.1534/g3.116.032300)
Supplement: Supplemental Material [file supp_6_10_3139__index.html]

Interallelic Transcriptional Enhancement as an in Vivo Measure of Transvection in Drosophila melanogaster — Supplemental Material 

# Interallelic Transcriptional Enhancement as an *in Vivo* Measure of Transvection in *Drosophila melanogaster*

## Supplemental Material for Noble, Dolph, and Supattapone, 2016 *et al.*, 2016

**Files in this Data Supplement:**

- Figure S1 - Interallelic enhancement of UAS-PrP expression occurs at a different locus using a different GAL4 driver. (.pdf, 115 KB)
