# Supplementary material for: Interallelic Transcriptional Enhancement as an in Vivo Measure of Transvection in Drosophila melanogaster
Source: G3 (Bethesda). 2016 Aug 2;6(10):3139–48. doi: 10.1534/g3.116.032300 (PMC5068936; doi:10.1534/g3.116.032300)
Supplement: Supplemental Material [file supp_g3.116.032300_FigureS1.pdf]

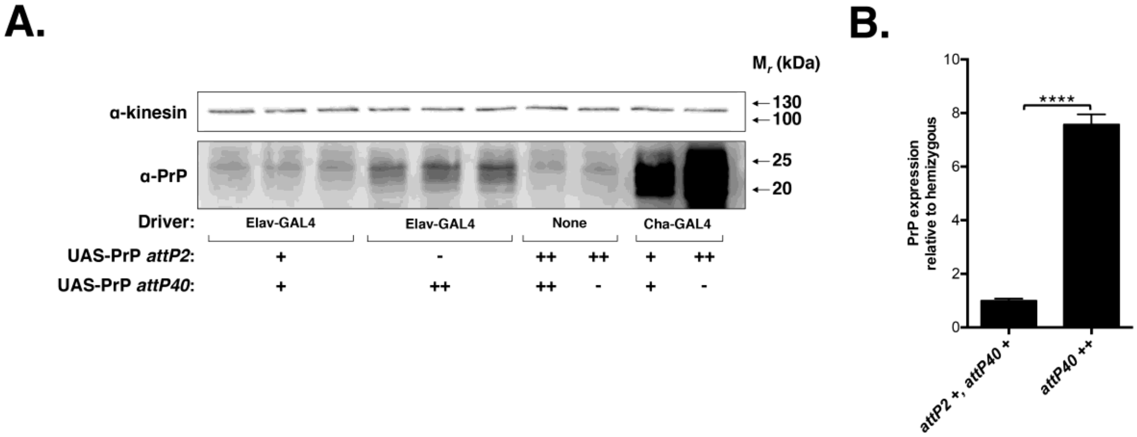

Figure S1: Interallelic enhancement of UAS-PrP expression occurs at a different locus using a different GAL4 driver. UAS-PrP inserted at *attP2* and/or *attP40* was expressed throughout the *Drosophila* nervous system, driven by Elav-GAL4 or Cha-GAL4. (*A*) Western blot of fly head homogenates (+ = hemizygous, ++ = homozygous at the indicated locus). Each lane represents a biological replicate. (*B*) Densitometry of Western blot signals from Elav-GAL4 lanes in panel *A*. PrP signals were normalized to the kinesin loading control and then to the non-allelic *attP2*, *attP40* UAS-PrP line. Error bars in part *B* represent standard deviations, with n = 3 for all samples; **** $p < 0.0001$.
